# Epidemiological situation of leprosy in a province in China: a long time to diagnosis and a high rate of deformity

**DOI:** 10.1186/s12889-020-09933-6

**Published:** 2020-11-25

**Authors:** Qing-Ping Zhang, Ge Li, Chao Li, Zhao-Xing Lin, Ping Chen

**Affiliations:** 1 Shaanxi Provincial Institute for Endemic Disease Control, Xi’an, 710003 China; 2 Xi’an Jiaotong University, Xi’an, 710061 Shaanxi China; 3 Department of Orthopedics, Xijing Hospital, the Fourth Military Medical University, West Road 169, Xi’an, Changle, 710032 Shaanxi Province China

**Keywords:** Leprosy, *Mycobacterium leprae*, China, Epidemiology

## Abstract

**Background:**

This epidemiological study aimed to analyse both the distribution and characteristics of leprosy in an endemic province in Northwest China.

**Methods:**

The medical records of leprosy patients in the province of Shaanxi, China, from 1998 to 2018 were collected from the Chinese Leprosy Management Information System (LEPMIS). Epidemiological variables were analysed in this study.

**Results:**

A total of 477 new cases were diagnosed between 1998 and 2018 in this region. The average annual detection rate was 0.070/100,000 population, and the average annual prevalence was 0.305/100,000 population. The mean age of the newly diagnosed patients was 46.7 years, and the ratio of males to females was 2.5:1. There were 399 cases (83.6%) of multibacillary (MB) leprosy. One hundred forty-eight patients (31.0%) had grade 2 disability. The mean diagnosis time for new cases was 62.0 months.

**Conclusion:**

This epidemiological study showed that the characteristics of newly diagnosed leprosy cases in our province were a long time to diagnosis and a high rate of deformity, indicating the need for actions focusing on early diagnosis and treatment and strengthening the detection of leprosy in low-prevalence areas.

## Background

Leprosy, which is caused by *Mycobacterium leprae*, is a chronic infectious disease [1] that mainly results in injuries to the skin and peripheral nerves. It is prevalent worldwide, and has been prevalent in China for more than 2000 years. Although there is no vaccine, leprosy treatment is effective, especially when leprosy is diagnosed early. However, the global leprosy prevention and treatment strategy has not undergone major revisions [2], and the focus is still on the early detection of cases and timely regular combined chemotherapy [3]. The long disease course, high deformity rate [4], and the cooccurrence other medical problems may result in social issues, such as low quality of life and low happiness index due to discrimination [5]. Since the founding of the People’s Republic of China in 1949, our government has carried out a great deal of public health work [6] and implemented a large number of effective leprosy prevention and control measures [7]. Measures include the detecting of socially active leprosy patients early, administering multidrug therapy (MDT) in a timely manner, terminating transmission, and controlling \ the epidemic to the maximum extent possible [8].

In 2018, the incidence rate of leprosy in Shaanxi Province, China, fell below 1/100,000 population, which is the cut-off value for the definition of leprosy elimination in China [9]. However, there are still sporadic new cases. As an important part of the Silk Road Economic Belt and the twenty-first-Century Maritime Silk Road (B&R), our province covers a total area of 205,800 km^2^ and has a resident population of 38.64 million (December 2018). Due to different regional and population distributions, to explore the epidemic trend and characteristics of leprosy in this province, we retrospectively reviewed leprosy cases between 1998 and 2018 and investigated the characteristics of diagnostic time and rate of grade 2 disability due to leprosy, and we provide a basis for the effective prevention and control of leprosy in areas with low leprosy rates.

## Methods

### Diagnosis standard

The diagnosis of new or recurrent leprosy was based on the Leprosy Prevention Manual for Primary Care Physicians (PCPs) and Leprosy Diagnosis Standard WS291–2018 [10]. A recurrent case was defined as a patient who completed a prescribed course of treatment and had no lasting symptoms but had clinical, bacterial, or histopathological evidence of disease recurrence after achieving clinical cure (clinical inactivity) [10]. Cases were classified as having MB or PB (paucibacillary) leprosy according to the degree of skin-smear positivity [11]. The time to diagnosis was defined as the time interval between disease onset and the diagnosis of leprosy. Disability classification was based on the Disability Classification Standard for Leprosy, WHO, 1998 [12]. In 1988, the WHO Expert Committee on Leprosy substantially simplified the disability grading system into a three-grade (0,1 and 2) classification system [13]. Patients with grade 0 disability have no loss of sensation, no visible deformity and no eye problems due to leprosy. Patients with grade 1 disability have loss of sensation or eye problems due to leprosy present but no visible deformity. Patients with grade 2 disability have visible deformity or severe visual impairment. In 1998, the Committee endorsed this grading system with the amendment that lagophthalmos, iridocyclitis and corneal opacities be included in the grade 2 criteria [12].

### Data sources

All leprosy cases (including new, recurrent and prevalent cases) were collected from the Leprosy Prevention and Control Management Information System in China (LEPMIS). LEPMIS was designed by the Chinese government in 2010 and reports the data of not only newly diagnosed patients and those undergoing treatment but also those who have been cured and achieved lifelong management, thus comprising a detailed and comprehensive database. LEPMIS not only includes related information about disease discovery and diagnosis but also information about all aspects of leprosy management. In terms of medical record management, it not only helps with case management but also effectively monitors the management of patients and their close contacts. The data of leprosy patients from 1998 to 2009 were collected from paper files and were uploaded to LEPMIS in 2010. Administrative regions in China are divided into four levels: province, region, county, and village. In Shaanxi Province, there were 11 regions in 2018 (i.e., the Xi’an region). Seventy-two counties and 30 districts belonged to these regions. County and district administrative levels are equivalent. Population data was obtained from the Shaanxi Statistical Yearbook [14].

### Statistical analysis

A new and recurrent leprosy case database was established in EXCEL 2010. Data were analysed using the *χ*^*2*^test for trend. Statistical analyses were performed using SPSS Statistics 19.0 (IBM Corp., Armonk, NY, USA).

## Results

### Overall situation and change trend

Between 1998 and 2018, 542 leprosy cases were reported in Shaanxi Province, with an annual detection rate of 0.070/100,000 population, as shown in Table 1. Among them, 477 were new cases, with a new case detection rate of 0.061/100,000 population. Sixty-five leprosy cases were recurrent cases, with a recurrence rate of 0.008/100,000 population. The number of current leprosy cases in this area is 2372, with an annual prevalence rate of 0.305/100,000 population. The annual new case detection rate dropped from 0.078/100,000 population in 1998 to 0.029/100,000 population in 2018, with a significant decreasing trend (*χ*^*2*^ = 86.85, P<0.01). The annual recurrence rate dropped from 0.014/100,000 population in 1998 to 0.000/100,000 population in 2018, with a significant decreasing trend (*χ*^*2*^ = 16.14, *p* < 0.01). The annual detection rate dropped from 0.092/100,000 population in 1998 to 0.029/100,000 population in 2018, with a significant decreasing trend (*χ*^*2*^ = 112.99, *p* < 0.01). The annual prevalence rate dropped from 0.309/100,000 population in 1998 to 0.134/100,000 population in 2018, with a significant decreasing trend (*χ*^*2*^ = 671.15, p < 0.01), as shown in Table 1.

**Table 1:** Demographic characteristics of incident and prevalent cases in Shaanxi province from 1988 to 2018

Year	Total population of Shaanxi, × 100,000	Prevalent cases				Detected cases		New cases				Relapse case	
		N	Prevalence rate, per 100,000	Sex ratio, M/F	Average age, M ± S, years	N	Annual detection rate, per 100,000	New cases	New case rate, per 100,000	Sex ratio of new cases, M/F	Average age of new cases, M ± S, years	Relapse cases	Relapse rate, per 100,000
1998	359.6	111	0.309	5.8	38.4 ± 12.2	33	0.092	28	0.078	2.11	42.7 ± 10.5	5	0.014
1999	361.8	96	0.265	13.0	46.6 ± 13.9	15	0.042	11	0.030	2.67	43.6 ± 11.9	4	0.011
2000	360.5	115	0.319	3.4	40.4 ± 12.2	35	0.097	34	0.094	1.83	42.9 ± 11.8	1	0.003
2001	359.4	143	0.398	2.3	45.3 ± 14.8	46	0.128	42	0.117	1.63	43.8 ± 12.9	4	0.011
2002	359.3	152	0.423	5.0	46.6 ± 14.4	33	0.092	31	0.086	5.20	45.2 ± 15.0	2	0.006
2003	359.7	150	0.417	7.0	43.8 ± 11.2	23	0.064	21	0.058	6.00	43.0 ± 10.7	2	0.006
2004	360.2	161	0.447	3.4	45.7 ± 12.1	31	0.086	24	0.067	2.43	42.0 ± 9.3	7	0.019
2005	367.2	170	0.463	2.7	47.8 ± 13.5	41	0.112	35	0.095	3.38	46.2 ± 13.5	6	0.016
2006	374.1	162	0.433	2.0	47.8 ± 14.3	47	0.126	40	0.107	1.86	47.0 ± 13.6	7	0.019
2007	375.3	170	0.453	2.8	50.2 ± 14.7	46	0.123	39	0.104	3.33	48.9 ± 14.4	7	0.019
2008	375.6	157	0.418	2.6	45.9 ± 16.4	20	0.053	16	0.043	3.00	44.5 ± 16.4	4	0.011
2009	347.7	129	0.341	3.7	48.0 ± 16.2	26	0.069	20	0.053	3.00	43.0 ± 14.2	6	0.016
2010	377.8	102	0.270	1.9	47.7 ± 17.2	23	0.061	21	0.056	2.00	46.9 ± 17.0	2	0.005
2011	373.2	106	0.284	1.9	44.8 ± 16.2	26	0.070	24	0.064	1.67	43.7 ± 16.2	2	0.005
2012	389.1	86	0.221	1.8	47.8 ± 12.0	16	0.041	16	0.041	1.67	47.2 ± 12.4	0	0.000
2013	375.0	69	0.184	2.7	51.0 ± 13.5	11	0.029	10	0.027	2.33	51.0 ± 14.1	1	0.003
2014	374.3	64	0.171	3.7	45.4 ± 14.7	14	0.037	13	0.035	3.33	45.3 ± 13.1	1	0.003
2015	388.2	59	0.152	2.5	46.6 ± 15.0	14	0.037	12	0.032	3.00	36.7 ± 11.9	2	0.005
2016	365.3	61	0.167	3.0	48.9 ± 10.1	15	0.041	14	0.038	3.67	49.2 ± 10.7	1	0.003
2017	365.4	57	0.156	2.2	48.3 ± 11.4	16	0.044	15	0.041	2.00	49.5 ± 10.8	1	0.003
2018	388.1	52	0.134	1.8	49.3 ± 12.5	11	0.029	11	0.029	1.75	49.2 ± 12.5	0	0.000
Total	–	–	–	–	–	542	1.397	477	1.229	2.46	46.3 ± 13.3	65	0.167

*Abbreviations*: *M/F* Males/Females, *M ± S* Mean ± Standard deviation

### Population distribution

#### Sex distribution

Among 477 newly diagnosed leprosy patients, 339 were males and 138 were females, with a male-to female ratio of 2.46:1; there was a significant difference between the sexes (*χ*^*2*^ = 304.17, *p* < 0.01), as shown in Table 1.

#### Age distribution

Between 1998 and 2018, the average age of the newly diagnosed leprosy patients in Shaanxi was 46.3 years old, and the average age of newly diagnosed patients increased from 42.7 years old in 1998 to 49.2 years old in 2018, as shown in Table 1. During this period, 2 cases in 14-year-old paediatric patients were detected (1, Ankang region, 2004; 1: Hanzhong region, 2007).

### Regional distribution

A total of 542 new and recurrent cases occurred in local residents, and in 2018, 10 counties or districts in Shaanxi reported newly diagnosed cases. In 2018, 28 counties or districts reported existing cases, which were mainly distributed in southern Shaanxi, including Hanzhong, Ankang, and Shangluo. Leprosy cases were scattered in Guanzhong, and no cases were reported in northern Shaanxi for many years. By the end of 2018, only one county (Yangxian County) in Shaanxi had not reached the basic leprosy elimination index (1/100,000) stipulated by the Ministry of Health [9], China; the prevalence rate in Yangxian County was 1.29/100,000 population.

### Types of cases and disability

#### Case types

Among 477 newly discovered cases, 399 cases (83.6%) were MB, and 78 cases (16.4%) were PB leprosy, as shown in Table 2. The ratio of MB:PB leprosy was 5.12:1. No significant difference was found in the MB:PB leprosy ratio of between 1998 and 2018 (*χ*^*2*^ = 0.002, *P* > 0.05). A significant difference was found in the MB:PB leprosy ratio between newly detected and recurrent cases (*χ*^*2*^ = 192.92, *p* < 0.01).

**Table 2:** Diagnostic and clinical characteristics of new leprosy cases in Shaanxi from 1998 to 2018

Year	Cases	MB	PB	MB proportion (n%)	Average diagnostic delay, m	Cases with grade 2 disability	The proportion with grade 2 disability (n%)
1998	28	25	3	89.29	64.17	3	10.71
1999	11	10	1	90.91	62.11	2	18.18
2000	34	30	4	88.24	64.12	7	20.59
2001	42	38	4	90.48	62.14	22	52.38
2002	31	25	6	80.65	58.26	6	19.35
2003	21	17	4	80.95	43.80	4	19.05
2004	24	23	1	95.83	80.83	6	25.00
2005	35	26	9	74.29	43.94	11	31.43
2006	40	30	10	75.00	112.38	15	37.50
2007	39	31	8	79.49	97.97	14	35.90
2008	16	12	4	75.00	33.44	3	18.75
2009	20	19	1	95.00	58.65	11	55.00
2010	21	16	5	76.19	47.85	4	19.05
2011	24	20	4	83.33	47.21	7	29.17
2012	16	14	2	87.50	64.25	5	31.25
2013	10	8	2	80.00	52.45	4	40.00
2014	13	11	2	84.62	54.62	6	46.15
2015	12	11	1	91.67	71.42	4	33.33
2016	14	9	5	64.29	88.50	8	57.14
2017	15	14	1	93.33	46.50	3	20.00
2018	11	10	1	90.91	47.60	3	27.27
Total	477	399	78	83.65		148	31.03

*Abbreviations*: *MB* multibacillary, *PB* Paucibacillary

#### Disability

Of 477 newly diagnosed cases, 148 (31.03%) were associated with grade 2 disabilities. The rate of disability increased from 10.71% in 1998 to 27.27% in 2018.

### Time to diagnosis in new cases

The average time to diagnosis in 477 newly detected cases was 62.0 (43.8–112.4) months according to the LEPMIS. However, the average time to diagnosis was shorter in 2018 than in 1998 (47.6 vs 64.0 months), as shown in Table 2.

## Discussion

Between 1925 and 2018, 11,789 leprosy patients were diagnosed with leprosy in Shaanxi Province; 10,270 patients were cured, and 502 patients experienced relapse (data from LEPMIS). The prevalence rates of leprosy in over 95% of the regions, counties, and districts in Shaanxi achieved control, with rates below 1/100,000 population, reaching our national target of eliminating leprosy. According to the above data, 477 newly diagnosed leprosy cases and 65 recurrent cases were reported in Shaanxi between 1998 and 2018, with 542 cases in total. The annual detection rate dropped from 0.092/100,000 population in 1998 to 0.029/100,000 population in 2018. The detection rate and prevalence rate of leprosy showed impressively significant downward trends over the past 20 years, indicating that we have achieved good results in the prevention and control of leprosy.

In 477 newly diagnosed patients, the male-female ratio was 2.46:1, which was basically consistent with those in previous reports from other areas in China [15, 16]. This result was consistent with previous results obtained from other regions of the country [17]. Male patients still accounted for a relatively high proportion of the newly diagnosed cases each year, which may be related to different genetic susceptibilities between the sexes [18]. However, this ratio in China was different from those in some other countries around the world [19]. This may be because females undergo more skin consultations than males, and males may be more frequently exposed to leprosy bacilli than females due to behavioural and cultural factors [20]. Cultural, lifestyle, and genetic differences between races may be related to a difference in incidence rates, but to date, no studies have been published on this topic. It would be interesting to explore this aspect.

The average age of the newly diagnosed patients was 45.2 years old, which was relatively stable over the study period. In terms of newly diagnosed cases, the age distribution in different years was similar. However, the average age of prevalent cases increased over time [21]. The Leprosy Control Handbook [22] notes that the average age of patients tends to rise as the incidence of leprosy declines (the prevalence of leprosy is controlled). This supports the relatively low rates of leprosy in Shaanxi. However, this may also be related to the long time to diagnosis [23], suggesting that the detection of new leprosy patients in Shaanxi still needs improvement. In terms of current leprosy cases, the average age of 21 years was relatively high, which may be related to the large number of MB leprosy cases in our province and the long MDT course. Disability degree is an indicator of the ability to diagnose and monitor leprosy in health service departments, and patients classified with grade 1 or grade 2 disability had a long time to diagnosis or monitoring failure [24]. This suggests that we should pay attention to the treatment and rehabilitation of current leprosy cases. From 1998 to 2018, only two children under 15 years old were diagnosed with leprosy, which may be related to the long incubation period and/or the low transmission rate and the relatively low prevalence in Shaanxi. In terms of regional distribution, leprosy epidemics mainly occurred in southern Shaanxi and the Central Shaanxi Plain [25]. These areas are also areas with historically high leprosy incidence rates. The source of infection has not been completely and effectively controlled, suggesting that we should continue to improve prevention and control work in these areas.

A total of 83.6% of the new cases were MB leprosy, and 16.4% were PB leprosy, with a ratio of 5.1:1, which was higher than that in related reports from other regions in China [26]. This indicated that the number of MB cases was too high and that there were potential risks. MB leprosy patients have more severe symptoms, have more complications and require a longer course of MDT than PB leprosy patients. In Shaanxi, the proportion of MB patients was relatively higher than that in other provinces. This high proportion resulted in leprosy patients in Shaanxi having more severe symptoms, having more complications and being cured at an older age on average than PB leprosy patients. However, previous studies have shown that the type ratio increases in areas with a low leprosy prevalence [27], which may indicate that leprosy in Shaanxi was in a low epidemic state and under effective control. The type ratio of newly detected cases between 1998 and 2018 was relatively high (83.7%). The grade 2 disability rate in new cases in Shaanxi between 1998 and 2018 was approximately 31.0%, which may be related to the prolonged time to diagnosis and few newly detected cases. The average time to diagnosis in newly diagnosed patients was 46 months, which was much higher than those reported in relevant literature in some other areas in China, such as Guangdong Province [21], indicating that leprosy cases in Shaanxi were associated with a longer time from onset to diagnosis. This situation may be related to latent symptoms and atypical clinical manifestations in the early stage, little attention to patients, lack of primary prevention and treatment experience, etc. [28], suggesting that our province should further carry out leprosy monitoring and strengthen early detection and diagnosis.

To improve the diagnostic time and reduce the deformity rate in Shaanxi Province, we should take advantage of China’s health system reform, comprehensively improve the LEPMIS, strengthen the training of dermatologists, vigorously promote community health consultations for leprosy, and actively carry out early leprosy diagnosis technology research.

## Conclusion

Although leprosy prevention and control in Shaanxi has achieved remarkable results and the leprosy epidemic has been maintained at a relatively low level for a long time, the prevalence of leprosy was still relatively high in some areas, and the incidence of disability in new leprosy patients was still high in some counties. The time to diagnosis still needs improvement. To achieve the goal of eliminating morbidity caused by leprosy, we should further strengthen the detection, treatment, and management of leprosy cases; focus on how to shorten the period between symptom onset and diagnosis; and reduce the rate of leprosy-associated disability.

## Data Availability

The data that support the findings of this study are available from the Health Commission of Shaanxi Province; however, restrictions apply regarding the availability of these data, which were used under a license for the current study, and the data are not publicly available. The data may available from the authors upon reasonable request and with permission from the Health Commission of Shaanxi Province. Please contact the corresponding author: C Li: 397564825@qq.com.

## Abbreviations

LEPMIS: Leprosy Management Information System in China
MB: Multibacillary
PB: Paucibacillary
B&R: The Silk Road Economic Belt and the twenty-first-Century Maritime Silk Road
PCPs: Primary Care Physicians
MDT: Multidrug Therapy
